# Investigating the Combination of Deep Learning for Channel Estimation and Power Optimization in a Non-Orthogonal Multiple Access System

**DOI:** 10.3390/s22103666

**Published:** 2022-05-11

**Authors:** Mohamed Gaballa, Maysam Abbod, Ammar Aldallal

**Affiliations:** 1 Department of Electronic & Electrical Engineering, Brunel University London, Uxbridge UB8 3PH, UK; maysam.abbod@brunel.ac.uk; 2 Department of Telecommunication Engineering, Ahlia University, Manama P.O. Box 10878, Bahrain; aaldallal@ahlia.edu.bh

**Keywords:** deep learning, LSTM, NOMA, optimization, KKT conditions

## Abstract

In a non-orthogonal multiple access (NOMA) system, the successive interference cancellation (SIC) procedure is typically employed at the receiver side, where several user’s signals are decoded in a subsequent manner. Fading channels may disperse the transmitted signal and originate dependencies among its samples, which may affect the channel estimation procedure and consequently affect the SIC process and signal detection accuracy. In this work, the impact of Deep Neural Network (DNN) in explicitly estimating the channel coefficients for each user in NOMA cell is investigated in both Rayleigh and Rician fading channels. The proposed approach integrates the Long Short-Term Memory (LSTM) network into the NOMA system where this LSTM network is utilized to predict the channel coefficients. DNN is trained using different channel statistics and then utilized to predict the desired channel parameters that will be exploited by the receiver to retrieve the original data. Furthermore, this work examines how the channel estimation based on Deep Learning (DL) and power optimization scheme are jointly utilized for multiuser (MU) recognition in downlink Power Domain Non-Orthogonal Multiple Access (PD-NOMA) system. Power factors are optimized with a view to maximize the sum rate of the users on the basis of entire power transmitted and Quality of service (QoS) constraints. An investigation for the optimization problem is given where Lagrange function and Karush–Kuhn–Tucker (KKT) optimality conditions are applied to deduce the optimum power coefficients. Simulation results for different metrics, such as bit error rate (BER), sum rate, outage probability and individual user capacity, have proved the superiority of the proposed DL-based channel estimation over conventional NOMA approach. Additionally, the performance of optimized power scheme and fixed power scheme are evaluated when DL-based channel estimation is implemented.

## 1. Introduction

A non-orthogonal multiple access (NOMA) system has been classified as promoting a multiple access structure for future wireless systems to boost system throughput and spectral efficacy. NOMA could utilize the present resources more effectively by resourcefully benefiting from the users’ channel environments and providing numerous users with distinct quality of service (QoS) demands. NOMA enables several users to achieve simultaneous arrival to the same time-frequency block by superpositioning them in the code or power domains [1]. The concept of NOMA is established on that the user with a weak channel condition can be combined with the user with a good channel condition in the same time slot and on the same allocated subcarrier to ensure that the bandwidth block could be effectively exploited [2].

In the NOMA scheme, receiver equipment will receive the multiplexing of symbols from different users in the system; therefore, elimination of interference from other users is necessary for coordinated decoding.

Generally, multiuser detection (MUD) in NOMA can be managed through SIC, which was carried out in the power domain. In the SIC technique, symbols from several users are decoded consecutively based on the allocated signal power and the channel state information (CSI) [3]. Complete realization of CSI or channel status for individual users is challenging, since pilot symbols that are utilized in channel estimation may interfere with symbols from other users, thus influencing the performance of classical channel estimation procedures, such as minimum mean square error and least square estimators [4]. Machine learning (ML) algorithms have the capability to adapt to variations in channel between user and base station (BS); therefore, ML is regarded as a strong contender for future radio networks [5].

### 1.1. Related Works

In [6], the authors introduced DL-based detector for the multiuser downlink OFDM-NOMA system. The authors mainly depended on pilot signals for the channel information, and according to these pilot responses, a DL based joint channel estimation and symbol detection was achieved without additional processing for channel estimation. The simulation’s outcomes revealed that the proposed DL scheme outperforms the conventional SIC-based detector. On the other hand, the proposed scheme needed to be initially trained offline for different channel conditions and the simulation results were presented in terms of BER only.

In [7], the authors suggested a deep learning framework to perform signal recovery in the MIMO-NOMA system when the Rayleigh fading channel is considered. The proposed technique can simultaneously carry out the channel estimation process and signal detection. Simulations were conducted for the proposed DL scheme, and the results were compared with the conventional SIC procedure in terms of the symbol error rate (SER) and throughput. According to the simulation results, the proposed DL scheme can address channel impairment, but the examined NOMA cell was limited for two users and an offline training stage was also required. Also, the DNN training phase needed two components, the received signal, and the labels, which were used as supervised data to help the DNN to optimize the parameters.

In [8], the authors proposed a data-driven deep learning estimator for time- and frequency-selective channels. The proposed algorithm was designed such that a pre-training scheme and pilot symbols were utilized as inputs for the DNN to attain a desired initialization, which can further enhance the performance of the DL estimator. The DNN was trained offline in both the pre-training and training stages, while in the testing stage, the channels could be dynamically tracked by the DNN with only pilots identified, and then the transmitted symbols were detected. The performance of a DL estimator with different numbers of layers was investigated and the numerical results demonstrated that the proposed DL estimator outperformed the standard channel estimator in terms of efficiency and robustness.

In [9], a deep learning approach was employed to estimate the downlink channel and to reduce the training overhead in a fog radio access network. The Gated Recurrent Unit (GRU) was utilized to learn the hidden correlations among the channel matrices from different users, and a bidirectional GRU was also employed to further improve the estimation performance. Simulation results were provided to demonstrate the performance gains, but the examined performance metrics were limited to the loss function and mean square error.

Based on the deep learning (DL) algorithm, the authors in [10] introduced a sliding window Gated Recurrent Unit (GRU) channel estimator to acquire knowledge for the time-varying Rayleigh fading channel. Interleaver and channel coding schemes were merged with the proposed sliding window estimator to further enhance system performance. The simulation results proved the ability of the suggested procedure to follow the channel in a reliable way and achieve better mean square error (MSE). Moreover, the sliding window-based GRU estimator was examined with different numbers of pilot symbols, and the robustness against the variations in the channel characteristics was analyzed.

In [11], the authors went to conclude that DL algorithm can be utilized in signal detection for uplink analysis in NOMA network. The authors proposed a DL approach to characterize the complex channel parameters, where restricted Boltzmann machines (RBM) were implemented as a pre-training phase for the original input sequence for the network. The proposed learning scenario based on LSTM layer could track the environment statistics automatically via offline learning and an iterative support detection procedure was suggested to identify the transmitted symbols. Performance analysis for the proposed DL scheme was evaluated merely in terms of the sum data rate and block error rate.

In [12], a pilot-aided receiver structure was presented for an uplink single input, multi output (SIMO) NOMA system, which incorporated a combined channel estimation and signal detection scheme for random channels. The authors brought together a deep learning model with SIC detection structure to minimize the learnable parameters. Furthermore, signal detection accuracy improvement and noise interference reduction were achieved by adding noise and interference elimination factors at the SIC detection stage. The simulation results indicated that BER performance based on the proposed DL scheme was more acceptable than the traditional MMSE procedure and the complexity of the receiver was diminished.

In [13], the authors proposed a semi-blind mutual detection scheme-based DL to distinguish users’ symbols in the co-operative NOMA system. The proposed method was capable of detecting the signal without the need for further channel estimation process since it could achieve a simultaneous detection on the basis of pilot responses. The DL model was trained offline over a Rayleigh fading channel and the trained network was deployed in the online detection phase. In addition, the trained model was inspected using Rician and Na-agami-m fading channels and simulation outcomes proved the capability of the proposed scheme in outperforming conventional detectors.

In [14], the authors examined deep neural network (DNN) for combined channel estimation and signal detection in an OFDM system. This approach considered OFDM system and fading channel as a black box and the presented DNN network is trained offline using simulated data. The simulation results revealed that the proposed DL approach had the capability to learn and investigate the complicated attributes of the wireless channels. In addition, the results of the DL approach proved its dominance over conventional methods when fewer pilot symbols were utilized, and cyclic prefix was ignored.

### 1.2. Research Gap and Motivation

Based on the previous works, many deep neural networks (DNN) approaches have been proposed explicitly to address the issues associated with channel state information (CSI), channel estimation, and signal detection. To the best of authors’ knowledge, there has been no study that has investigated the combination between Deep learning (DL)-based channel estimation algorithm and the optimal power allocation scheme for multiuser detection in a downlink non-orthogonal multiple access (NOMA) system in fading channels. Most of the works are managing the issues of power optimization and deep learning-based channel estimation separately.

In addition, many of the proposed DNN approaches for channel estimation require pre-training to obtain the appropriate DNN weights initialization, which may lead to an increase of the number of hidden layers with a huge number of neurons in each layer. In order to enhance the aforementioned schemes, this work proposes a framework that examine the integration between DNN-based channel estimation and the optimum power allocation scheme in a multiuser NOMA system and then inspect the system performance. Furthermore, in our proposed system structure, we manage to eliminate the need for a pre-training stage, minimize the number of DNN layers, and minimize the number of pilot symbols, and at the same time, we can realize a notable improvement in network convergence.

### 1.3. Contributions to Knowledge

The detailed contributions of this work are outlined as follows:

- In this work, a framework is proposed that highlights how the channel estimation-based Deep Learning (DL) and power optimization scheme are jointly utilized for multiuser (MU) detection in the PD-NOMA system.
- A structured and mathematical analysis is introduced to derive a non-complex analytical form for the optimum power coefficient for each user on the basis of maximizing the sum rates in a downlink NOMA system.
- An optimized power scheme and fixed power scheme are both evaluated and compared when the proposed Deep Learning-based channel estimation scheme is implemented.
- To validate the efficiency of the proposed Deep Learning channel estimation scheme, the DNN model is inspected using a Rayleigh fading channel and Rician fading channel.
- As a benchmark comparison, we have also conducted the simulation environment related to the work that consider the DL for joint channel estimation and signal detection and compared it with our proposed DL scheme. The simulation results emphasized that reliability can be guaranteed by our proposed DL channel estimation scheme even when cell capacity is increased.
- In addition, different from the aforementioned works, in our proposed DL channel estimation algorithm, we implement a minimum number of DNN layers and a minimum number of pilot symbols to achieve a remarkable improvement in system performance. Moreover, no additional interference cancelation or noise elimination factors are utilized on the receiver side.

The rest of this paper is organized as follows. Section 2 presents the system model. In Section 3, the characterization for the optimization problem is introduced. In Section 4, the optimization analysis is discussed in detail. In Section 5, RNN and LSTM DL schemes are described. Section 6 discusses the LSTM and NOMA framework. The channel estimation algorithm is summarized in Section 7. In Section 8, the simulation environment is described. In Section 9, the simulation results are discussed. Finally, the conclusions and future work are drawn in Section 10.

## 2. System Model

The downlink NOMA system is examined in this section, where the base station (BS) and users come across various channel gains. In this system, the NOMA cell is considered, where one BS with a single antenna is assumed to serve three users concurrently and every user equipment also has a single antenna. Typically, in NOMA scheme each user receives the combined signal sent from BS that comprise a target signal and interfering signal sent through the same time-frequency block. Consequently, multiplexing a number of signals using distinct power levels is essential to distinguish the signals and to reinforce the SIC procedure at the receiver side [15]. In PD-NOMA, users that are characterized by good channel environments are usually allocated low power, while users with poor channel conditions can share high power levels.

Users are labeled in accordance with their fading channel and the separation from base station. In the examined cell, the nearby device is indicated as near user and the device at the edge of the cell is viewed as far user. In this work, we assume that we have three users in the cell and Rayleigh fading channel is considered with zero mean. Hence, the fading channel for each user can be mathematically characterized as follows, for the near user $h_{n}\sim \left(0,d_{n}^{-k}\right)$, for the middle user $h_{m}\sim \left(0,d_{m}^{-k}\right)$, and for the far user $h_{f}\sim \left(0,d_{f}^{-k}\right)$, where $h_{i}$ denotes the fading channel between the BS and the user *i* and *k* represents the path loss exponent [16].

In this paper, Additive White Gaussian Noise (AWGN) is assumed at the receiver side of each user, and the noise power is indicated as $\sigma^{2}$. Without loss of generality, it can be assumed that ${\left|h_{n}\right|}^{2}>{\left|h_{m}\right|}^{2}>{\left|h_{f}\right|}^{2}$. Total power transferred from BS to all devices in the cell is specified as $P_{t}$. In the NOMA system, the receiver at each user has the ability to carry out SIC to eliminate signals associated to other users with poor channel environments. On the other hand, symbols from users with good channel conditions could not be removed and treated as interference. In the downlink scenario, the BS can send the superposition coded signal x that can be expressed as [12,16]:

(1) $$x=\sqrt{P_{t}}\left(\sqrt{\alpha_{n}}x_{n}+\sqrt{\alpha_{m}}x_{m}+\sqrt{\alpha_{f}}x_{f}\right)$$

where $\alpha_{n}$, $\alpha_{m}$, and $\alpha_{f}$ are the power coefficients allocated to the near user, middle user, and far user individually. Likewise, $x_{n}$, $x_{m}$, and $x_{f}$ denote the desired symbols concerned to near, middle, and far user, respectively. Hence, the signal received at far user can be represented as [17]:

(2) $$y_{f}=xh_{f}+z_{f}$$

where $h_{f}$ represent the fading channel among BS and far user, while $z_{f}$ represents AWGN noise component at far user with zero mean and $\sigma^{2}$ variance. The far user is usually described by poor channel condition, and their particular signal $x_{f}$ can be assigned additional power by BS compared to other users. Thus, according to SIC scheme, the far user can directly decode their own signal $x_{f}$ from received signal $y_{f}$. The received signal for a far user device can be easily represented as [17]:

(3) $$y_{f}=\sqrt{P_{t}}\left(\sqrt{\alpha_{n}}x_{n}+\sqrt{\alpha_{m}}x_{m}+\sqrt{\alpha_{f}}x_{f}\right)h_{f}+z_{f}\;y_{f}=\sqrt{P_{t}\alpha_{f}}x_{f}h_{f}+\sqrt{P_{t}}\left(\sqrt{\alpha_{m}}x_{m}+\sqrt{\alpha_{n}}x_{n}\right)h_{f}+z_{f}$$

The first term in (3) represents the required signal for a far user, while the second term denotes the interference term from middle and near users. Based on Equation (3), the possible rate for far user could be expressed as [18]:

(4) $$R_{f}=\log_{2}\left(1+\frac{{\left|h_{f}\right|}^{2}P_{t}\alpha_{f}}{{\left|h_{f}\right|}^{2}P_{t}\left(\alpha_{n}+\alpha_{m}\right)+\sigma^{2}}\right)$$

Likewise, the attainable bit rate for middle user can also be expressed as:

(5) $$R_{m}=\log_{2}\left(1+\frac{{\left|h_{m}\right|}^{2}P_{t}\alpha_{m}}{{\left|h_{m}\right|}^{2}P_{t}\left(\alpha_{n}\right)+\sigma^{2}}\right)$$

Typically, the near user has good channel condition along with BS; thus, their signal $x_{n}$ is assigned low power, and therefore, the received signal for the near user can be shown as:

(6) $$y_{n}=xh_{n}+z_{n}\;y_{n}=\sqrt{P_{t}\alpha_{n}}x_{n}h_{n}+\sqrt{P_{t}}\left(\sqrt{\alpha_{m}}x_{m}+\sqrt{\alpha_{f}}x_{f}\right)h_{n}+z_{n}$$

The first term in Equation (6) represents the near user expected signal, while the second term denotes the interfering term from middle and far users. On the other hand, it can be observed from Equation (6) that the interfering term is predominant due to the additional power that can be assigned to the far user. Therefore, at the near user side, SIC is performed, where immediate decoding for far user signal $x_{f}$ is accomplished, then removed from the composite signal. Next, the middle user signal $x_{m}$ is decoded and removed from the remaining signal. Finally, the near user achieved rate $R_{n}$ can be expressed as [18]:

(7) $$R_{n}=\log_{2}\left(1+\frac{{\left|h_{n}\right|}^{2}P_{t}\alpha_{n}}{\sigma^{2}}\right)$$

## 3. Optimization Problem

The aim here is to maximize the sum rate for users in NOMA system on the basis of optimizing the power factors for each user in accordance with the channel conditions. The sum of the above-mentioned possible rates for N users downlink NOMA network can be written as follows [19,20]:

(8) $$R_{sum}=\overset{N}{\underset{k=1}{\sum }}\log_{2}\left(1+\frac{{\left|h_{k}\right|}^{2}P_{t}\alpha_{k}}{{\left|h_{k}\right|}^{2}\sum_{j=1}^{k-1}P_{t}\alpha_{j}+\sigma^{2}}\right)$$

In the proposed system, the objective function and the constraints considered for the optimization problem can be clarified in the following sections.

### 3.1. Power Constraint

The allocated power for each user in the cell is a percentage of the overall power $P_{t}$ transferred from base station; thus, the allocated power fraction for each user equipment must complies with [21]:

(9) $$\overset{N}{\underset{x=1}{\sum }}\alpha_{x}\leq 1$$

where $\alpha_{x}$ is the power portion for the *x*th ser in the N-user NOMA cell.

### 3.2. QoS Constraints

To enhance user fairness, it is assumed that the weak user in each cell has a QoS requirement, which implies that a minimum rate $R_{min}$ needs to be guaranteed corresponding to the optimization problem considered, which can be expressed as follows [22]:

(10) $$\log_{2}(1+\delta_{n})\geq R_{min}$$

where $\delta_{n}$ is the SINR for *n*th user and $R_{min}$ is the minimum transmission rate required in the system [23]. This constraint can be simplified in many ways, suppose we have $R_{m\to k}$ which is the rate of user *k* to detect the signal of user *m*, where 1≤k≤m and $R_{m}=R_{min}$. When user k is not able to detect the message of user m with rate $R_{min}$, this can be indicated as $R_{m\to k}<R_{min}$ [23]. The complement of this event can be formulated as follows:

(11) $$\frac{{\left|h_{k}\right|}^{2}P_{t}\alpha_{m}}{{\left|h_{k}\right|}^{2}P_{t}\sum_{i=1}^{m-1}\alpha_{i}+\sigma^{2}}>(2^{R_{min}}-1)$$

where $\alpha_{i}$ is the power factor for *i*th user in the system. By dividing both the numerator and denominator of left-hand side of Equation (11) by the noise power $\sigma^{2}$, Equation (11) can be reformulated as:

(12) $${\left|h_{k}\right|}^{2}\rho \left(\alpha_{m}-(2^{R_{min}}-1)\overset{m-1}{\underset{i=1}{\sum }}\alpha_{i}\right)>(2^{R_{min}}-1)$$

where ρ represent the signal power to noise power ratio. Equation (12), also declares that in order to satisfy the minimum transmission rate and avert that *m*th user being in outage, the following condition must be achieved:

(13) $$\alpha_{m}>\left(2^{R_{min}}-1\right)\overset{m-1}{\underset{i=1}{\sum }}\alpha_{i}$$

where $\alpha_{m}$ is the power assigned for *m*th user in the system.

### 3.3. Sum Rate

On the basis of the aforementioned constraints in Equations (9) and (10) and sum rate representation and the fact that there is a single antenna at the BS and user equipment, the standard optimization problem can be normally expressed as follows [21,22]:

(14) $$\underset{\alpha }{\max}\;R_{sum}=\overset{N}{\underset{k=1}{\sum }}\log_{2}\left(\frac{{\left|h_{k}\right|}^{2}P_{t}\sum_{j=1}^{k-1}\alpha_{j}+\sigma^{2}+{\left|h_{k}\right|}^{2}P_{t}\alpha_{k}}{{\left|h_{k}\right|}^{2}P_{t}\sum_{j=1}^{k-1}\alpha_{j}+\sigma^{2}}\right)$$

such that:

$$\begin{array}{l} \sum_{x=1}^{N}\alpha_{x}\leq 1\\ \log_{2}(1+\delta_{n})\geq R_{min}\\ \alpha_{k}\geq 0\;\forall k=1,2,\ldots ,N \end{array}$$

## 4. Optimization Analysis

In this part, the optimization analysis is realized with regards to three users in the NOMA system and the objective function can simply reformulated as follows [24]:

(15) $$\underset{\alpha }{\max}\;R_{Sum}=R_{n}+R_{m}+R_{f}$$

$$\begin{array}{l} S.t.\\ \;(2^{R_{min}}-1)-{\left|h_{k}\right|}^{2}\rho \left(\alpha_{m}-(2^{R_{min}}-1)\sum_{i=1}^{m-1}\alpha_{i}\right)\leq 0\\ \;\alpha_{n}+\alpha_{m}+\alpha_{f}-1\leq 0\\ \;\alpha_{n},\;\alpha_{m},\alpha_{f}\;\geq 0 \end{array}$$

where m=2, 3 and $R_{min}$ is the lowest rate required in the system. According to the analysis above, the constraints can also be represented as follows:

(16) $$C_{1}\left(\alpha \right)=\alpha_{n}+\alpha_{m}+\alpha_{f}-1$$

(17) $$C_{2}\left(\alpha \right)=(2^{R_{min}}-1)-\rho {\left|h_{f}\right|}^{2}\left(\alpha_{f}-(2^{R_{min}}-1)(\alpha_{n}+\alpha_{m}\right)$$

(18) $$C_{3}\left(\alpha \right)=(2^{R_{min}}-1)-\rho {\left|h_{m}\right|}^{2}\left(\alpha_{m}-(2^{R_{min}}-1)(\alpha_{n}\right)$$

The constraints $C_{1}\left(\alpha \right),\;C_{2}\left(\alpha \right)\;\&\;C_{3}\left(\alpha \right)$ are linear in terms of α, then $C_{1}\left(\alpha \right),\;C_{2}\left(\alpha \right)\;\&\;C_{3}\left(\alpha \right)$ are convex. Hence, $\nabla R_{Sum}\left(\alpha \right)\;\&\;\nabla^{2}R_{Sum}\left(\alpha \right)$ need to be calculated [21]. Initially, we can find the first derivative for $R_{Sum}\left(\alpha \right)$ in Equation (14) with respect to each of the power coefficients $\alpha_{n}$, $\alpha_{m}$, and $\alpha_{f}$. After some mathematical processing, $\nabla R_{Sum}\left(\alpha \right)$ can be represented as follows [24,25]:

(19) $$\begin{array}{ll} \frac{\partial R_{Sum}}{\partial \alpha_{n}}=\frac{1}{ln2} & (\frac{|h_{n}{|}^{2}P_{t}}{|h_{n}{|}^{2}P_{t}\alpha_{n}+\sigma^{2}}-\frac{(|h_{m}{|}^{2}P_{t}{)}^{2}\alpha_{m}}{\left(|h_{m}{|}^{2}P_{t}(\alpha_{n}+\alpha_{m})+\sigma^{2})(|h_{m}{|}^{2}P_{t}(\alpha_{n})+\sigma^{2}\right)}\\ & -\frac{{\left(|h_{f}{|}^{2}P_{t}\right)}^{2}\alpha_{f}}{\left(|h_{f}{|}^{2}P_{t}(\alpha_{n}+\alpha_{m}+\alpha_{f})+\sigma^{2})(|h_{f}{|}^{2}P_{t}(\alpha_{n}+\alpha_{m})+\sigma^{2}\right)}) \end{array}$$

(20) $$\begin{array}{cc} \frac{\partial R_{Sum}}{\partial \alpha_{m}}=\frac{1}{ln2} & (\frac{{\left|h_{m}\right|}^{2}P_{t}}{{\left|h_{m}\right|}^{2}P_{t}(\alpha_{n}+\alpha_{m})+\sigma^{2}}\\ & -\frac{{\left({\left|h_{f}\right|}^{2}P_{t}\right)}^{2}\alpha_{f}}{\left({\left|h_{f}\right|}^{2}P_{t}\left(\alpha_{n}+\alpha_{m}+\alpha_{f}\right)+\sigma^{2}\right)\left({\left|h_{f}\right|}^{2}P_{t}\left(\alpha_{n+}\alpha_{m}\right)+\sigma^{2}\right)}) \end{array}$$

(21) $$\frac{\partial R_{Sum}}{\partial \alpha_{f}}=\frac{1}{ln2}\left(\frac{{\left|h_{f}\right|}^{2}P_{t}}{{\left|h_{f}\right|}^{2}P_{t}(\alpha_{n}+\alpha_{m}+\alpha_{f})+\sigma^{2}}\right)$$

At this point, a general formula can be derived for the first derivative of the objective function in terms of α [24,25]:

(22) $$\begin{array}{cc} \frac{\partial R_{Sum}\left(\alpha \right)}{\partial \alpha_{i}}= & \frac{1}{ln2}\;\;\left(\frac{{\left|h_{i}\right|}^{2}P_{t}}{{\left|h_{i}\right|}^{2}P_{t}\sum_{j=1}^{i}\alpha_{j}+\sigma^{2}}\right)-\\ & \frac{1}{ln2}\sum_{k=1}^{N-i}\{\left(\frac{{\left({\left|h_{\left(i+k\right)}\right|}^{2}P_{t}\right)}^{2}\alpha_{i+k}}{\left({\left|h_{\left(i+k\right)}\right|}^{2}P_{t}\sum_{j=1}^{i+k}\alpha_{j}+\sigma^{2}\right)}\right)\times \left(\frac{1}{\left({\left|h_{\left(i+k\right)}\right|}^{2}P_{t}\sum_{j=1}^{i+k-1}\alpha_{j}+\sigma^{2}\right)}\right)\} \end{array}$$

The second derivative of the objective function $R_{Sum}\left(\alpha \right)$ with respect to each of the power coefficients $\alpha_{n}$, $\alpha_{m}$, and $\alpha_{f}$ can also be derived as follows:

(23) $$\begin{array}{cc} \frac{\partial^{2}R_{Sum}}{\partial \alpha_{n}^{2}}=-\frac{1}{ln2} & \left\{(\frac{{\left(|h_{n}{|}^{2}P_{t}\right)}^{2}}{{\left(|h_{n}{|}^{2}P_{t}\alpha_{n}+\sigma^{2}\right)}^{2}}\right)\\ & -(\frac{{\left(|h_{m}{|}^{2}P_{t}\right)}^{3}\alpha_{m}\;[\;2(|h_{m}{|}^{2}P_{t}\alpha_{n}+\sigma^{2})+|h_{m}{|}^{2}P_{t}\alpha_{m}]}{{\left(|h_{m}{|}^{2}P_{t}(\alpha_{n}+\alpha_{m})+\sigma^{2}\right)}^{2}\;{\left(|h_{m}{|}^{2}P_{t}(\alpha_{n})+\sigma^{2}\right)}^{2}})\\ & -(\frac{{\left(|h_{f}{|}^{2}P_{t}\right)}^{3}\alpha_{f}[2(|h_{f}{|}^{2}P_{t}(\alpha_{n}+\alpha_{m})+\sigma^{2})+|h_{f}{|}^{2}P_{t}\alpha_{f}]}{{\left(|h_{f}{|}^{2}P_{t}(\alpha_{n}+\alpha_{m}+\alpha_{f})+\sigma^{2}\right)}^{2}\;{\left(|h_{f}{|}^{2}P_{t}(\alpha_{n}+\alpha_{m})+\sigma^{2}\right)}^{2}})\} \end{array}$$

(24) $$\begin{array}{ll} \frac{\partial^{2}R_{Sum}}{\partial \alpha_{n}^{2}}=\frac{1}{ln2} & \left\{(\frac{{\left(|h_{m}{|}^{2}P_{t}\right)}^{2}}{{\left(|h_{n}{|}^{2}P_{t}(\alpha_{n}+\alpha_{m})\;+\sigma^{2}\right)}^{2}}\right)\\ & -(\frac{{\left(|h_{f}{|}^{2}P_{t}\right)}^{3}\alpha_{f}[\;2(|h_{f}{|}^{2}P_{t}(\alpha_{n}+\alpha_{m})+\sigma^{2})+|h_{f}{|}^{2}P_{t}\alpha_{f}]}{{\left(|h_{f}{|}^{2}P_{t}(\alpha_{n}+\alpha_{m}+\alpha_{f})+\sigma^{2}\right)}^{2}\;{\left(|h_{f}{|}^{2}P_{t}(\alpha_{n}+\alpha_{m})+\sigma^{2}\right)}^{2}})\} \end{array}$$

(25) $$\frac{\partial^{2}R_{Sum}}{\partial \alpha_{f}^{2}}=-\frac{1}{ln2}\;\;\left\{\left(\frac{{\left({\left|h_{f}\right|}^{2}P_{t}\right)}^{2}}{{\left({\left|h_{f}\right|}^{2}P_{t}(\alpha_{n}+\alpha_{m}+\alpha_{f})+\sigma^{2}\right)}^{2}}\right)\right\}$$

A general formula can also be found for the second derivative of the objective function in terms of α as follows:

(26) $$\begin{array}{c} \frac{\partial^{2}R_{Sum}\left(\alpha \right)}{\partial \alpha_{i}{}^{2}}=-\frac{1}{ln2}\;\;\{\left(\frac{{\left({\left|h_{i}\right|}^{2}P_{t}\right)}^{2}}{{\left({\left|h_{i}\right|}^{2}P_{t}\sum_{j=1}^{i}\alpha_{j}+\sigma^{2}\right)}^{2}}\right)\\ -\sum_{k=1}^{N-i}\{\left(\frac{{\left({\left|h_{\left(i+k\right)}\right|}^{2}P_{t}\right)}^{3}\alpha_{i+k}\left[2\left({\left|h_{\left(i+k\right)}\right|}^{2}P_{t}\sum_{j=1}^{k+i-1}\alpha_{j}+\sigma^{2}\right)+{\left|h_{\left(i+k\right)}\right|}^{2}P_{t}\alpha_{i+k}\right]}{{\left({\left|h_{\left(i+k\right)}\right|}^{2}P_{t}\sum_{j=1}^{i+k}\alpha_{j}+\sigma^{2}\right)}^{2}}\right)\\ \times \left(\frac{1}{{\left({\left|h_{\left(i+k\right)}\right|}^{2}P_{t}\sum_{j=1}^{i+k-1}\alpha_{j}+\sigma^{2}\right)}^{2}}\right)\}\} \end{array}$$

Based on the objective function, and $\frac{\partial R_{Sum}\left(\alpha \right)}{\partial \alpha_{i}}$ and $\frac{\partial^{2}R_{Sum}\left(\alpha \right)}{\partial \alpha_{i}{}^{2}}$, it can be proved that the objective function is concave and has a unique global maximum [23]. The Lagrange function and the KKT necessary conditions could be employed to achieve optimal power factors [25,26]:

(27) $$ℒ\left(\alpha_{n},\alpha_{m},\alpha_{f},\mu_{1},\mu_{2,},\mu_{3}\right)=R_{Sum}-\mu_{1}C_{1}\left(\alpha \right)-\mu_{2}C_{2}\left(\alpha \right)-\mu_{3}C_{3}\left(\alpha \right)$$

where $\mu_{1},\;\mu_{2},$ and $\mu_{3}$ represent Lagrange multipliers for the three user scenarios.

Optimality conditions can be written as follows:

(28) $$\frac{\partial R_{Sum}}{\partial \alpha_{n}}-\mu_{1}\frac{\partial C_{1}\left(\alpha \right)}{\partial \alpha_{n}}-\mu_{2}\frac{\partial C_{2}\left(\alpha \right)}{\partial \alpha_{n}}-\mu_{3}\frac{\partial C_{3}\left(\alpha \right)}{\partial \alpha_{n}}=0$$

(29) $$\frac{\partial R_{Sum}}{\partial \alpha_{m}}-\mu_{1}\frac{\partial C_{1}\left(\alpha \right)}{\partial \alpha_{m}}-\mu_{2}\frac{\partial C_{2}\left(\alpha \right)}{\partial \alpha_{m}}-\mu_{3}\frac{\partial C_{3}\left(\alpha \right)}{\partial \alpha_{m}}=0$$

(30) $$\frac{\partial R_{Sum}}{\partial \alpha_{f}}-\mu_{1}\frac{\partial C_{1}\left(\alpha \right)}{\partial \alpha_{f}}-\mu_{2}\frac{\partial C_{2}\left(\alpha \right)}{\partial \alpha_{f}}-\mu_{3}\frac{\partial C_{3}\left(\alpha \right)}{\partial \alpha_{f}}=0$$

Slackness conditions can be represented as follows:

(31) $$\mu_{1}\left(\alpha_{n}+\alpha_{m}+\alpha_{f}-1\right)=0$$

(32) $$\mu_{2}\left((2^{R_{min}}-1)-\rho {\left|h_{f}\right|}^{2}\left(\alpha_{f}-(2^{R_{min}}-1)(\alpha_{n}+\alpha_{m}\right)\right)=0$$

(33) $$\mu_{3}\left((2^{R_{min}}-1)-\rho {\left|h_{m}\right|}^{2}\left(\alpha_{m}-(2^{R_{min}}-1)(\alpha_{n}\right)\right)=0$$

Lagrange multipliers also need to satisfy the following:

(34) $$\mu_{1}\geq 0,\;\;\mu_{2}\geq 0,\;\;\mu_{3}\geq \;\;\;0$$

In the subsequent steps, Lagrange multipliers should be proved to be positive. This could be accomplished as follows:

(35) $$\frac{\partial C_{1}\left(\alpha \right)}{\partial \alpha_{n}}=\frac{\partial C_{1}\left(\alpha \right)}{\partial \alpha_{m}}=\frac{\partial C_{1}\left(\alpha \right)}{\partial \alpha_{f}}=1$$

(36) $$\frac{\partial C_{2}\left(\alpha \right)}{\partial \alpha_{n}}=\frac{\partial C_{2}\left(\alpha \right)}{\partial \alpha_{m}}=\rho {\left|h_{f}\right|}^{2}(2^{R_{min}}-1)$$

(37) $$\frac{\partial C_{3}\left(\alpha \right)}{\partial \alpha_{n}}=\rho {\left|h_{m}\right|}^{2}(2^{R_{min}}-1)\;$$

(38) $$\frac{\partial C_{2}\left(\alpha \right)}{\partial \alpha_{f}}=-\rho {\left|h_{f}\right|}^{2}$$

(39) $$\frac{\partial C_{3}\left(\alpha \right)}{\partial \alpha_{m}}=-\rho {\left|h_{m}\right|}^{2}$$

Based on Equations (35)–(39), this can be substituted in the optimality conditions for Lagrange as follows:

$$\begin{array}{c} \frac{\partial R_{Sum}}{\partial \alpha_{n}}-\mu_{1}\left(1\right)-\mu_{2}\rho {\left|h_{f}\right|}^{2}(2^{R_{min}}-1)\;-\mu_{3}\rho {\left|h_{m}\right|}^{2}(2^{R_{min}}-1)=0\\ \frac{\partial R_{Sum}}{\partial \alpha_{m}}-\mu_{1}\left(1\right)-\mu_{2}\rho {\left|h_{f}\right|}^{2}(2^{R_{min}}-1)-\mu_{3}\left(-\rho {\left|h_{m}\right|}^{2}\right)=0\\ \frac{\partial R_{Sum}}{\partial \alpha_{f}}-\mu_{1}\left(1\right)-\mu_{2}\left(-\rho {\left|h_{f}\right|}^{2}\right)-\mu_{3}\left(0\right)=0 \end{array}$$

Let $\beta 1=\rho {\left|h_{f}\right|}^{2}(2^{R_{min}}-1)$, $\beta 2=\rho {\left|h_{m}\right|}^{2}(2^{R_{min}}-1)$, $\gamma 1=\left(-\rho {\left|h_{f}\right|}^{2}\right)$, and $\gamma 2=\left(-\rho {\left|h_{m}\right|}^{2}\right)$. Therefore, the above written optimality conditions for Lagrange can be rewritten as:

(40) $$\frac{\partial R_{Sum}}{\partial \alpha_{n}}-\mu_{1}-\mu_{2}\;\beta 1-\mu_{3}\;\beta 2=0$$

(41) $$\frac{\partial R_{Sum}}{\partial \alpha_{m}}-\mu_{1}-\mu_{2}\;\beta 1-\mu_{3}\;\gamma 2=0$$

(42) $$\frac{\partial R_{Sum}}{\partial \alpha_{f}}-\mu_{1}-\mu_{2}\;\gamma 1=0$$

Based on Equation (40) and after few mathematical substitutions, the following expression can be written as:

(43) $$\left(\frac{\partial R_{Sum}}{\partial \alpha_{m}}-\frac{\partial R_{Sum}}{\partial \alpha_{f}}\right)-\left(\frac{\partial R_{Sum}}{\partial \alpha_{n}}-\frac{\partial R_{Sum}}{\partial \alpha_{f}}\right)\left(\frac{\gamma 2}{\beta 2}\right)=\mu_{2}\left(-\gamma 1+\beta 1+\left(\gamma 1-\beta 1\right)\left(\frac{\gamma 1}{\beta 1}\right)\right)$$

Performing a few mathematical analyses and based on the fact that ${\left|h_{n}\right|}^{2}>{\left|h_{m}\right|}^{2}>{\left|h_{f}\right|}^{2}$, we can simply prove that $\left(\frac{\partial R_{Sum}}{\partial \alpha_{m}}-\frac{\partial R_{Sum}}{\partial \alpha_{f}}\right)$ and $\left(\frac{\partial R_{Sum}}{\partial \alpha_{n}}-\frac{\partial R_{Sum}}{\partial \alpha_{f}}\right)$ are positive and the left-hand side of Equation (43) is positive. Furthermore, since $\left(\frac{\gamma 1}{\beta 1}\right)$ are negative scalar, the right-hand side of Equation (43) can be proved to be positive, which concludes that $\mu_{2}$ is positive. Additionally, Equation (42) can be reformulated as follows:

$$\frac{\partial R_{Sum}}{\partial \alpha_{f}}-\mu_{2}\;\gamma 1=\mu_{1}$$

where $\frac{\partial R_{Sum}}{\partial \alpha_{f}}$ is positive by inspection and $\left(\mu_{2}\gamma 1\right)$ is negative quantity; therefore, the left-hand side must be positive, which implies that $\mu_{1}$ is positive quantity.

Similarly, $\mu_{3}$ can be proved to be positive value. In accordance with the above-mentioned analysis, the examined constraints are feasible, and the closed form representation for the power factors can be determined from the slackness conditions as follows:

(44) $$\alpha_{n}+\alpha_{m}+\alpha_{f}=1$$

(45) $$(2^{R_{min}}-1)=\rho {\left|h_{f}\right|}^{2}\left(\alpha_{f}-(2^{R_{min}}-1)\left(\alpha_{n}+\alpha_{m}\right)\right)$$

(46) $$(2^{R_{min}}-1)=\rho {\left|h_{m}\right|}^{2}\left(\alpha_{m}-(2^{R_{min}}-1)\left(\alpha_{n}\right)\right)$$

For the following analysis, it can be assumed that $A_{1}=(2^{R_{min}}-1)$ and $A_{2}=\rho {\left|h_{f}\right|}^{2}$, $A_{3}=\rho {\left|h_{m}\right|}^{2}$, then Equation (45) can be written as $A_{1}=A_{2}\left(\alpha_{f}-A_{1}\left(\alpha_{n}+\alpha_{m}\right)\right)$ and Equation (46) can be rewritten as $A_{1}=A_{3}\left(\alpha_{f}-A_{1}\left(\alpha_{n}\right)\right)$.

Based on mathematical substitutions and arrangements, the closed form representation for each of the power coefficients can be derived as follows:

(47) $$\alpha_{f}=\left(\frac{A_{1}}{A_{2}}\right)\left(\frac{1+A_{2}}{1+A_{1}}\right)=\left(\frac{\left(2^{R_{min}}-1\right)}{2^{R_{min}}}\right)\left(1+\frac{1}{\rho {\left|h_{f}\right|}^{2}}\right)$$

(48) $$\begin{array}{l} \alpha_{m}=\left(\frac{1+\frac{1}{A_{3}}-\alpha_{f}}{1+\frac{1}{A_{1}}}\right)\;\\ \alpha_{m}=\left(\left(\frac{\left(2^{R_{min}}-1\right)}{2^{R_{min}}}\right)\left(1+\frac{1}{\rho {\left|h_{m}\right|}^{2}}\right)-{\left(\frac{2^{R_{min}}-1}{2^{R_{min}}}\right)}^{2}\left(1+\frac{1}{\rho {\left|h_{f}\right|}^{2}}\right)\right) \end{array}$$

(49) $$\begin{array}{l} \alpha_{n}=1-\left(\alpha_{m}+\alpha_{f}\right)\\ \alpha_{n}=\frac{\left(\frac{1}{A_{2}}\right)\left(\frac{1+A_{2}}{1+A_{1}}\right)-\left(\frac{A_{3}-A_{1}A_{2}}{A_{2}A_{3}}\right)}{1+A_{1}}\\ \alpha_{n}=\frac{1}{\left(2^{R_{min}}\right)}\left(\left(\frac{1+\rho {\left|h_{f}\right|}^{2}}{(2^{R_{min}})\rho {\left|h_{f}\right|}^{2}}\right)+\left(\frac{\left(2^{R_{min}}-1\right)}{\rho {\left|h_{m}\right|}^{2}}-\frac{1}{\rho {\left|h_{f}\right|}^{2}}\right)\right) \end{array}$$

## 5. RNNs and LSTM

**Figure 1:**
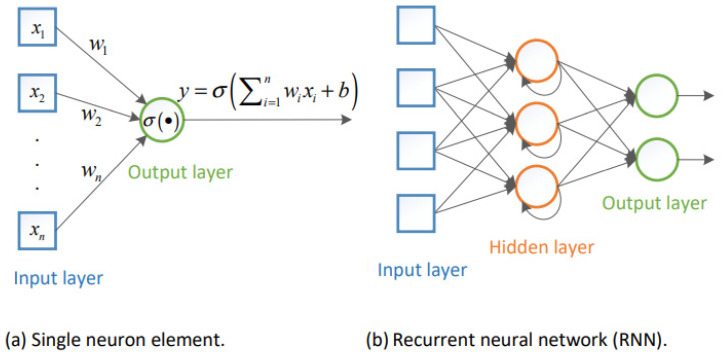
RNN network architecture.

Recurrent Neural Networks (RNNs) are regarded as a class of supervised learning procedures, and they can develop successive sequences for prediction and detection [27]. As shown in Figure 1, RNNs involve hidden layers composed of artificial neurons with feedback loops; therefore, they have dual inputs, i.e., the present and the recent previous response.

In RNNs, hidden layers are capable to act as storage for the network at a specific time; this structure enables the RNNs to handle the preceding data for a prolonged period of time; additionally, RNNs can represent time dependencies for any sequence with a lower numeral of neurons. On the other hand, traditional RNN based on backpropagation through time (BTT) experiences slow learning and a vanishing gradient problem [28].

Therefore, RNNs will not be the best candidate for signals that may be sent through fading channels that may disperse the signal and originate long-term dependencies among its samples [5]. Long short-term memory (LSTM) network, which is a one category of RNNs, is frequently used with sequences and time series data for categorization, where it can take advantage of time dependencies between sequences [5].

LSTM network can develop knowledge among time steps of the data sequence and manage the long-term dependency process of time series data. Based on their underlying design, the LSTM network includes LSTM cells, and each cell contains a set of gates that are capable of saving and gaining access to data over extended periods of time and of counteracting the error from backpropagation [5,28]. LSTM is able to receive a vector complex data, hence integrating the magnitude and phase parts of the received sequence concurrently. LSTM network can be considered as a proper selection to realize multiuser detection (MUD) and prediction when time series data are available [29].

## 6. Proposed DL Network Architecture and Framework

A framework that incorporates the LSTM model with NOMA system is discussed in this section, where the LSTM network is mainly utilized to perform training, updating, and prediction for the channel coefficients. Data-driven communication usually depends on empirical observations to determine the amount of LSTM cells in each layer and the numerals of LSTM layers that are needed in the implementation stage. Additionally, it is important to take in consideration that adding more LSTM layers may not offer a noticeable gain in learning phase, or it may not significantly affect the network convergence [30].

### 6.1. DL Network Architecture

**Figure 2:**
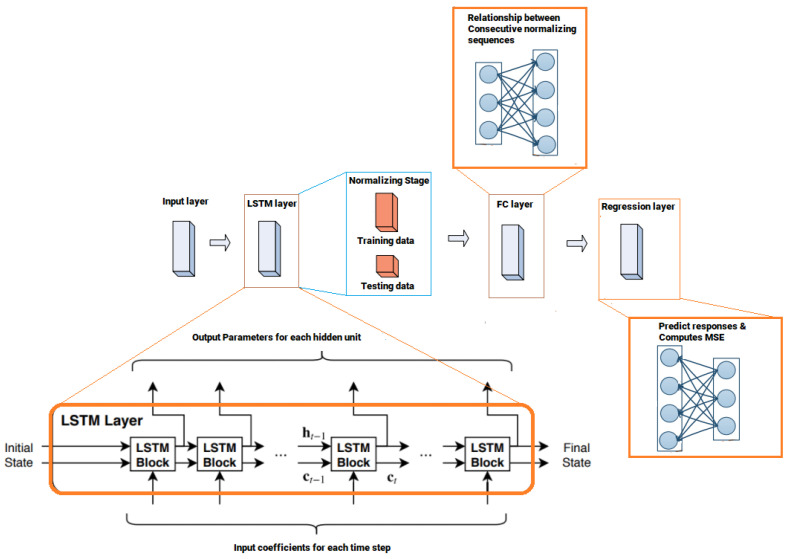
Architecture of the proposed DL network.

Figure 2 illustrates the architecture of the proposed DL network that consists of four layers, while each layer is supported with several neurons, and the weighted sum of each neuron will be the input to a nonlinear function. The dimension of each training sequence is indicated as ***L***, which is the length of the input layer. The input layer includes 128 neurons, where the inputs to the network are shifted to the subsequent layer with updated weight parameters.

In the second layer, one LSTM layer is implemented that includes 300 hidden units. The learnable weights of LSTM layer are the input weights ***W***, the recurrent weights ***R***, and the bias ***b***. The third layer is a fully connected layer that processes the outputs of the LSTM layer. A fully connected layer multiplies the input by a weight matrix ***W*** and then adds a bias vector ***b***. All neurons in a fully connected layer are connected to all the neurons in the preceding layer, and this layer bring together all of the characteristics and internal information gathered by the prior layers. In a DL-based LSTM network, the fully-connected layer behaves separately on each time step.

The last layer is the regression layer, which is responsible to improve the cell status, network weights, and biases. A regression layer can predict responses of a trained regression network and computes the *MSE* for regression tasks. Normalizing the responses mainly facilitates stabilizing and accelerating training of neural networks for regression. For a single observation, the mean square error can be calculated as [31]:

(50) $$MSE=\overset{r}{\underset{j=1}{\sum }}\frac{{\left(y_{Tj}-y_{Pj}\right)}^{2}}{r}$$

where r is the number of responses, $y_{Tj}$ is the target output, and $y_{Pj}$ is the predicted output at response j.

In case of sequence regression networks, the loss function can be represented as the half MSE of the predicted responses for each time step:

(51) $$loss=\frac{1}{2}\overset{L}{\underset{i=1}{\sum }}\overset{R}{\underset{j=1}{\sum }}\frac{{\left(y_{Tij}-y_{Pij}\right)}^{2}}{L}$$

where *L* is the sequence length.

### 6.2. LSTM Cell Structure and Mechanism

In LSTM cell, the output is generated based on the current input and the preceding cell state. In order to remember the previous cell state and determine if the prior state will be used or not, the LSTM cell consists of different types of gates, these gates are the forget gate, the input gate, and the output gate. In LSTM, there are two states, the cell state $C_{t-1}$, which is called internal memory where all information is stored, and the hidden state $h_{t-1}$, which is used for computing the output.

**Figure 3:**
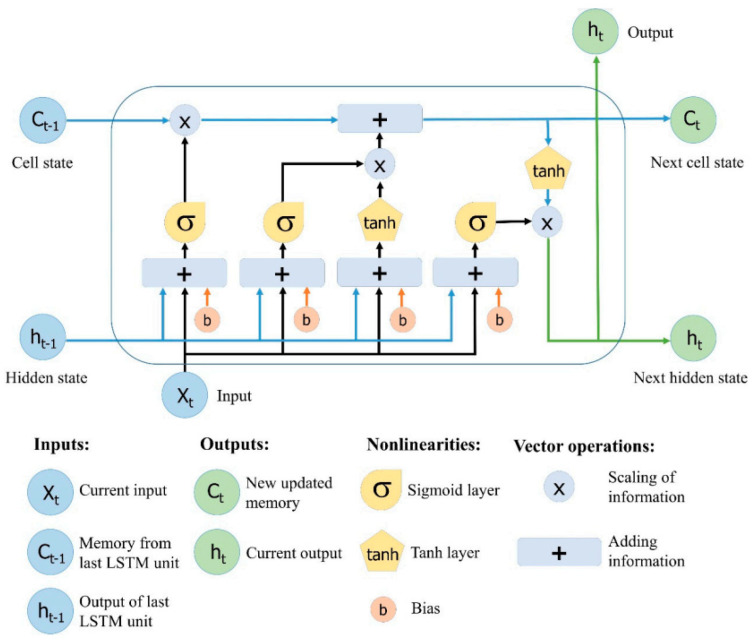
Internal structure of LSTM cell.

Figure 3 illustrates the internal structure of LSTM cell [32], where t represent the time instant, $x_{t}$ is the current input, and $h_{ti}$ represent the current output channel coefficients for user i at time t. In addition, $C_{t-1}$ represents the previous cell state, which is shifted from a hidden layer to the next every single iteration. At every time step, the LSTM cell can add up information or remove information from the cell state. LSTM cell can regulate these updates using several gates that can be briefly described as follows:

1. The forget gate is responsible for controlling the level of cell state that need to be reset: $f_{t}=\sigma \left(W_{f}x_{t}+R_{f}h_{t-1}+b_{f}\right)$;
2. The input gate is responsible for controlling the level of cell state that need to be updated: $i_{t}=\sigma \left(W_{i}x_{t}+R_{i}h_{t-1}+b_{i}\right)$;
3. The candidate state is responsible for adding information to the cell state: $g_{t}=tanh\left(W_{g}x_{t}+R_{g}h_{t-1}+b_{g}\right)$;
4. Updated cell state: $C_{t}=\left(C_{t-1}⊙f_{t}\right)+\left(i_{t}\;⊙g_{t}\right)$, where ⊙ is element-wise multiplication.
5. The output gate is responsible for controlling the level of cell state added to hidden state: $O_{t}=\sigma \left(W_{o}x_{t}+R_{o}h_{t-1}+b_{o}\right)$;
6. Estimated output coefficients: $h_{t}=O_{t}⊙tanh\left(C_{t}\right)$.

## 7. Channel Estimation-Based DL Algorithm

The transmitted frame involves data and pilot symbols. The applied channel model is assumed to be constant during one frame of pilot and data symbols and the channel coefficients are changing from one frame to another. In the proposed DL scheme, two major stages are implemented to achieve an effective DNN model for channel estimation. The first stage involves both training and testing, where the DNN model is trained and tested with the samples that are created based on variety of distinct Rayleigh channel coefficients [24,33]. In the second stage, the trained DNN model is utilized to explicitly predict the channel taps for each user and these estimated taps will be employed to recover the desired transmitted data symbols for each user.

### Dataset Generation

At the beginning of every training stage, the weights and bias values of LSTM layer are initialized, and during the training phase, weights and biases are modified according to a gradient descent procedure [5,30]. The distance of each user from the BS and the path loss exponent needs to be assigned in the dataset, so that the channel coefficients for each user are randomly generated to model the Rayleigh fading channel between the user and BS. Pilot symbols are generated at random and recognized at the BS and at the receiver side of each user.

On the basis of the initial channel factors generated and the pilot symbols, the size of the training and testing frames can be identified. The training model are carefully established based on the selected layers, the hidden units assigned for each layer, and the training parameters. In order to further accelerate and stabilize the training process of the training network, we choose to normalize the training data.

Throughout the training phase, the performance of the proposed DNN model-based LSTM layer is assessed using RMSE and loss functions. In the testing period, new fading coefficients will be randomly generated, such that these coefficients are not the same as those generated for training. Once the training and testing sequences are inspected using the training network, the trained model will be employed as online channel estimator for the users. The proposed DL technique for channel estimation can be outlined as shown in Algorithm 1.
**Algorithm 1** Proposed DL Channel Estimation scheme1.Initialize the learnable parameters of an LSTM layer (***W***, ***R***, ***b***), where ***W*** is the input weights, ***R*** is the recurrent weights, and ***b*** is the bias.  **Inputs**▪Number of Iterations.▪The distance of each user from the BS.▪Path loss exponent.▪Generate Initial random Rayleigh channel coefficients for each user based on the channel model.▪Generate known pilot symbols.▪Assign the initial power factors for each user.▪Identify the size of training sequence $\left(L_{T}\right)$ and size of testing sequence $\left(L_{S}\right)$.  **Procedure**2.Assign the training sequence $\left(Z_{T}\right)$, testing sequence $\left(Z_{S}\right)$**,** and the desired coefficients $\left(Z_{D}\right)$.3.Calculate the **mean** and **variance** $\left(\mu_{T},\;\sigma_{T}^{2}\right)$ for channel coefficients in training sequence at each iteration.4.Normalizing the training data $Z_{T}\to Z_{NT}$ based on $\left(\mu_{T},\;\sigma_{T}^{2}\right)$.5.Characterize the relationship between consecutive normalized training sequences as $Z_{NT\approx }(X_{NT},\;Y_{NT})$.6.Initialize the **training network** $\left(T_{net}\right)$ and assign the following:▪Number of layers.▪Number of hidden units.▪Training parameters.7.Use $\left(X_{NT},\;Y_{NT}\right)$ as inputs for the training model $(T_{net}$).8.**Predict** the output normalized coefficients $\left(Y_{NP}\right)$.9.**Update** the state of training model $(T_{net}$).   **For** i = 1: $L_{T}$   $\left[T_{net},\;Y_{NP}\right]$ = **Predict and Update state** $\left(T_{net},\;Z_{NT}\right)$   **End**10.Denormalize $Y_{NP}\to Y_{P}$.11.Calculate **RMSE** $\left(Z_{D}-Y_{P}\right)$ and **Loss** function.12.**Update** the state of $\left(T_{net}\;\right)$ and **reset** the values for $Y_{P}$.13.Normalize **testing** data $Z_{S}\to Z_{NS}$, using $\left(\mu_{T},\;\sigma_{T}^{2}\right)$.14.Use normalized testing data $\left(Z_{NS}\right)$ as inputs for updated trained network $(T_{net}$).    **For** i = 1: $L_{S}$    $\left[T_{net},\;Y_{NP}\right]$ = **Predict and Update state** $(T_{net},\;Z_{NS}$)    **End**    **Outputs**15.**Predicted** normalized channel coefficients $Y_{NP}$.16.Denormalize $Y_{NP}\to Y_{P}$.17.Calculate **RMSE** $\left(Z_{D}-Y_{P}\right)$ & **Loss** function.18.Estimate channel taps (trained DNN model, pilot symbols).

## 8. Simulation Environment

In this section, a description of the simulation settings and parameters is introduced. Our examined downlink NOMA cell contains one base station (BS) and three distinct users in which the BS and each user in the cell is supplied with one antenna. For the downlink NOMA scenario, the modulated signals are superimposed and transmitted by BS to the users through uncorrelated Rayleigh and Rician fading channels affected by additive white gaussian noise (AWGN), where the noise spectral density $N_{0}=-174$ dBm and path loss exponent is 4.

In this paper, simulations are conducted using MATLAB to simulate and to emphasize the following: first, to evaluate the effectiveness of embedding the proposed DL-based LSTM network to accurately estimate the channel parameters for each user in downlink NOMA cell. Second, to integrate the proposed DL channel estimation algorithm with the derived optimized power allocation scheme and compare it with the NOMA system when fixed power factors are considered along with the proposed DL scheme. Monte-Carlo simulations are conducted with $N=10^{6}$ iterations, and at the start of every single set of iterations, pilot symbols are generated at random and recognized at the BS and at the receiver side of each user. The main simulation parameters are summarized in Table 1.

In our simulation environment, we assume that the channel state information (CSI) is not available at the receiver side. Therefore, for the sake of comparison and in order to investigate the efficiency of the proposed DL algorithm, two methods for channel estimation are implemented at the receiver side for each user. The first method, which is the proposed scheme, uses DNN-based LSTM layer to estimate the desired channel coefficients. The gradient descent algorithm is applied in conjunction with LSTM layer, and the LSTM layer is attached to a fully connected layer, where each neuron in the former layer is fully connected to every neuron in the consequent layer. The second channel estimation scheme implemented at each receiver side is initiated based on the minimum mean square error (MMSE) [33]. The MMSE technique will be applied as a conventional channel estimation technique for each user in NOMA cell, and in the simulations results, we refer to the MMSE scheme as conventional NOMA, to clarify that users are using the MMSE procedure for estimating the channel coefficients before recovering the original signal.

Channel taps that are employed to model the Rayleigh fading wireless channel are generated on the basis of ITU channel models. Throughout the simulations, NOMA system parameters are employed on the basis of the long-term evolution (LTE) standard [34]. Both training and implementation phases are conducted online throughout the simulations, and the fading coefficients in the testing stage are generated such that these coefficients are not the same as in the training stage. At the end of the training stage, which includes training and testing data, the trained network will be utilized as online channel estimator for the users rather than the conventional NOMA scheme that use the MMSE procedure for channel estimation.

Initially, different power factors are allocated for each user according to their distance from the BS and the current channel gain. Power allocation coefficients $\alpha_{n}$, $\alpha_{m}$, and $\alpha_{f}$ are defined for near, middle, and far users, respectively. In the fixed power allocation (FPA) scenario, we can assign $\alpha_{f}=0.7$, $\alpha_{m}=0.2$, and $\alpha_{n}=0.1$. Alternatively, in the optimized power scheme, power factors are apportioned between users according to the analytical form derived earlier for each user in Section 4. The propagation distances for each user with respect to base station are initially assigned in the simulation files as follows $d_{f}=1000\;m$, $d_{m}=500\;m$, and $d_{n}=200\;m$. Quadrature phase shift keying QPSK is utilized as a modulation scheme for the data symbols and pilot sequences. The applied transmitted power mainly varies from 0 to 30 dBm.

## 9. Simulation Results and Discussion

**Figure 4:**
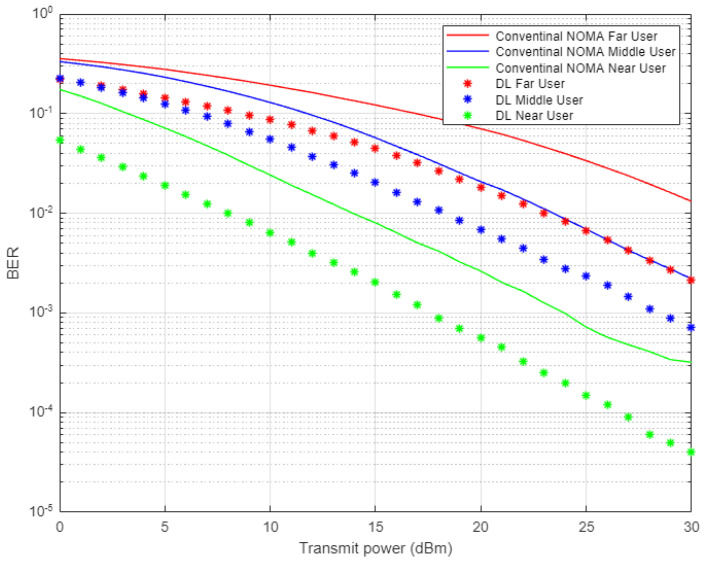
BER vs. power-based proposed DL-NOMA and conventional NOMA (Rayleigh Channel).

In Figure 4, the simulation results illustrate the comparison between the proposed DL scheme for channel estimation and conventional NOMA scheme that employs MMSE procedure for estimating the channel parameters. The estimated channel coefficients using both schemes will be used in signal recognition for far, middle, and near users and the simulation results are shown in terms of bit error rate (BER) versus transmitted power. All users in the NOMA cell-based DL channel estimation show sufficient improvement in lowering the bit errors compared to the conventional NOMA scenario, especially when the assigned power is increased. It can be noticed that for certain BER values, such as 10^−2^, the power saving achieved by DL scheme is approximately 5–8 dBm for far and middle users, while for the near user, the power saving is up to 4 dBm.

**Figure 5:**
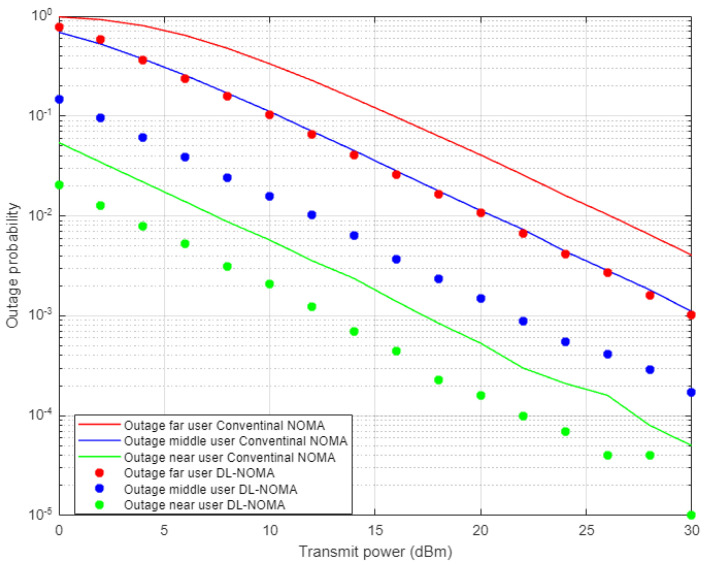
Outage probability vs. power-based DL and conventional NOMA (Rayleigh Channel).

Figure 5 demonstrates the results for outage probability metric versus transmitted power for the three examined users in NOMA system on the basis of DL and conventional NOMA channel estimator schemes. Far user and near user simulation results indicate an approximately 5 dBm improvement in power saving to achieve a certain outage probability (10^−3^) when DL-based channel estimation scenario is implemented compared to the conventional estimation scheme. Likewise, the middle user with the DL estimation scheme shows more enhancement in power saving compared to the MMSE procedure, by 5–7 dBm approximately, which proves the superiority of the proposed DL estimation scheme.

**Figure 6:**
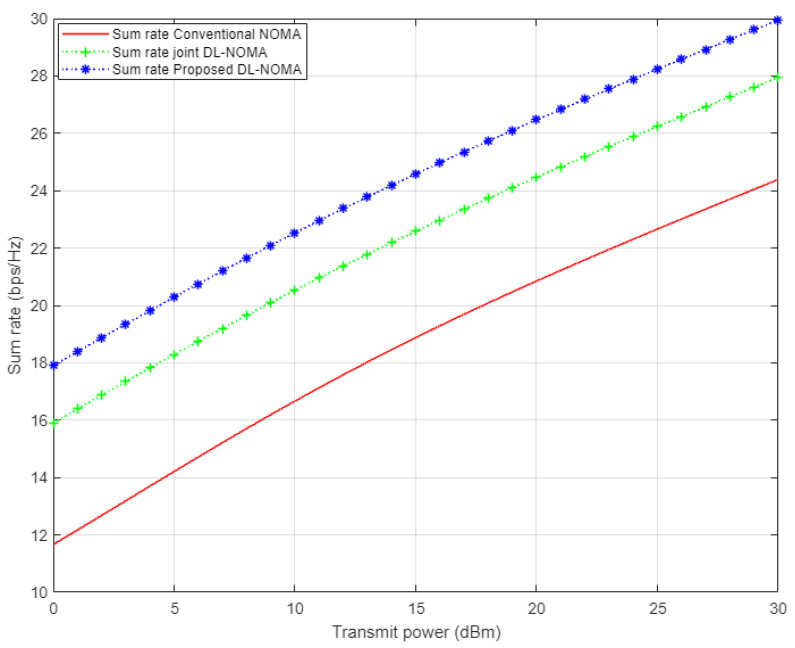
Sum rate vs. power for conventional NOMA, joint DL-NOMA, and proposed DL-NOMA (Rayleigh Channel).

Figure 6 displays the simulation results for the sum rate versus the transmitted energy for the three examined users in the NOMA cell. In this figure, three different channel estimation schemes are inspected, i.e., the proposed DL approach, conventional NOMA based on the MMSE scheme, and the DL algorithm for joint channel estimation and signal detection that was applied in [14]. Based on the simulation results, it can be clearly noticed that the proposed DL channel estimation scheme shows dominance over the conventional NOMA scenario, with 6 b/s/Hz approximately, and also indicates an improvement over the DL algorithm implemented in [14] by 2 b/s/Hz. These results verify the effectiveness of the proposed DL scheme in estimating the channel coefficients before being utilized in the decoding process.

**Figure 7:**
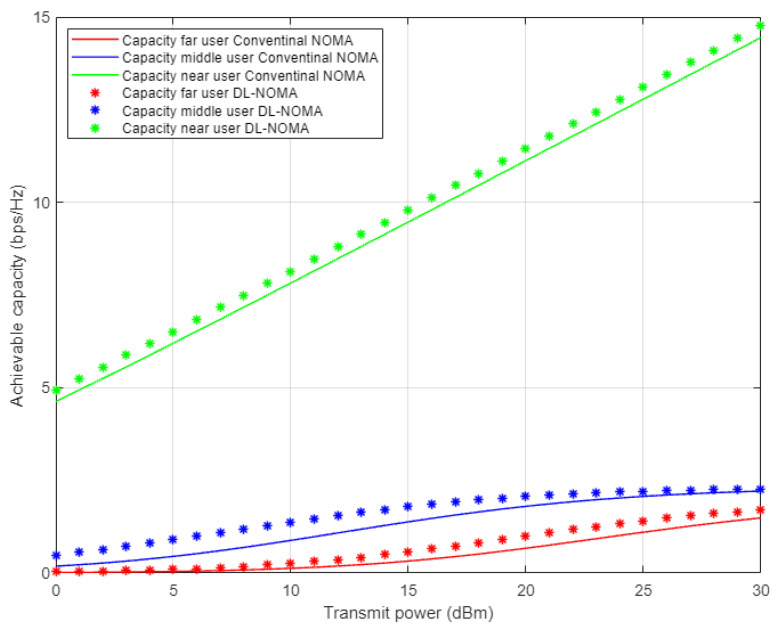
Individual capacity vs. power-for proposed DL-NOMA and conventional NOMA (Rayleigh Channel).

Figure 7 illustrates the simulation results for the individual capacity metric for each user in the NOMA cell when both the proposed DL-assisted channel estimation and conventional NOMA-based MMSE channel estimation schemes are employed. As expected, when power level starts to increase, the achieved capacity for the near user shows significant difference by at least 8 b/s/Hz approximately over far and middle users’ rates. This may be justified by the good channel condition for the near user compared to other users in the cell. Furthermore, the proposed DL approach still delivers noticeable enhancements with respect to other users, but with little impact especially for the far user, due to interference and weak channel conditions.

**Figure 8:**
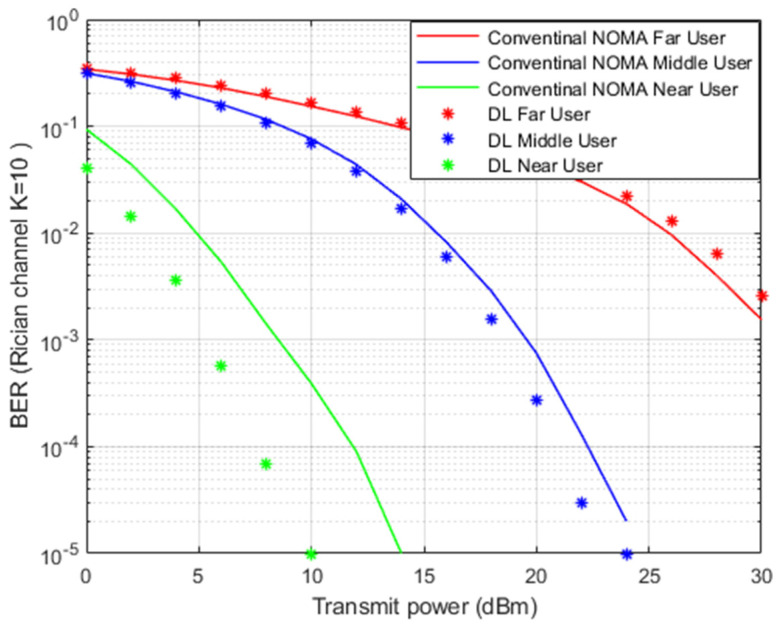
BER vs. power-based DL and conventional NOMA (Rician Channel).

Figure 8 illustrates the simulation results for BER versus transmitted power when the Rician channel is implemented. The proposed DL channel estimation scheme and conventional MMSE scheme will be further inspected by the Rician channel model.

Rician fading is a stochastic model for radio propagation, where the signal arrives at the receiver by several different paths, and hence, exhibits multipath interference. Rician fading occurs when one of the paths, typically a line of sight (LOS) signal or some strong reflection signals, is much stronger than the others. A Rician fading channel can be described by two parameters The first one is the Rician factor *K* defined as the ratio of the signal power in the line-of-sight component to the scattered power in other components. The other main parameter is Ω, which represents the total power from both paths and acts as a scaling factor to the distribution. In our simulation file for the Rician channel, we assign K = 10, sample rate = 9600 Hz, and maximum doppler shift = 100.

In Figure 8, simulation outcomes for near and middle users indicate a noticeable improvement in lowering the bit errors when the proposed DL scheme is applied compared to the conventional MMSE scenario. The near user shows a substantial improvement in terms of power saving due to the relaxed channel conditions and elimination of interference by the SIC method. In the far user situation, the impact of DL in tracking the channel parameters is limited due to the interference and weak channel conditions.

**Figure 9:**
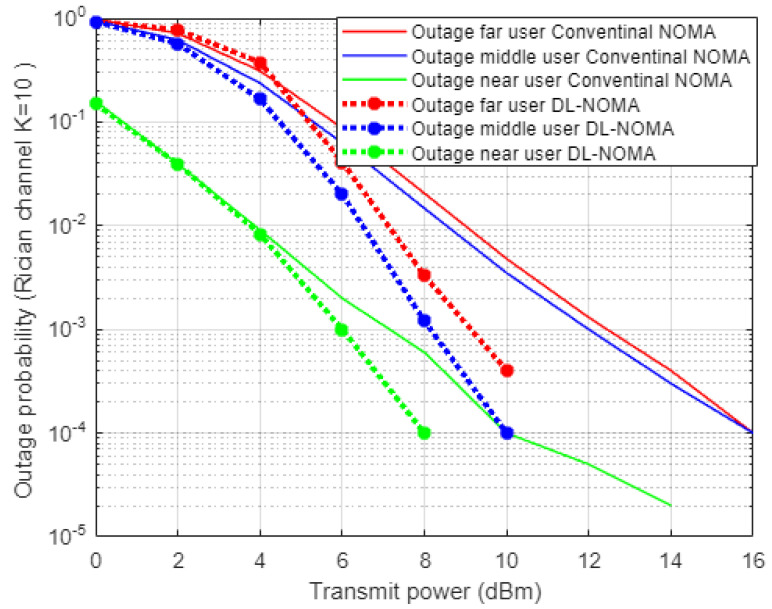
Outage probability vs. power-based DL and conventional NOMA (Rician channel).

Figure 9 demonstrates the simulation results for outage probability metric versus power transmitted when the Rician channel is considered, and both DL and MMSE channel estimation schemes are inspected. Far and middle users’ simulation results show an improvement within approximately 4 dBm of power saving to achieve a certain outage probability when the DL-based channel estimation scenario is conducted compared to the MMSE scheme. In terms of the near user, the simulation outcomes indicate that the proposed DL channel estimation scheme starts showing improvement regarding the outage probability when the power allocated to the near user is more than 4 dBm, which also proves the dominance of the proposed DL method.

**Figure 10:**
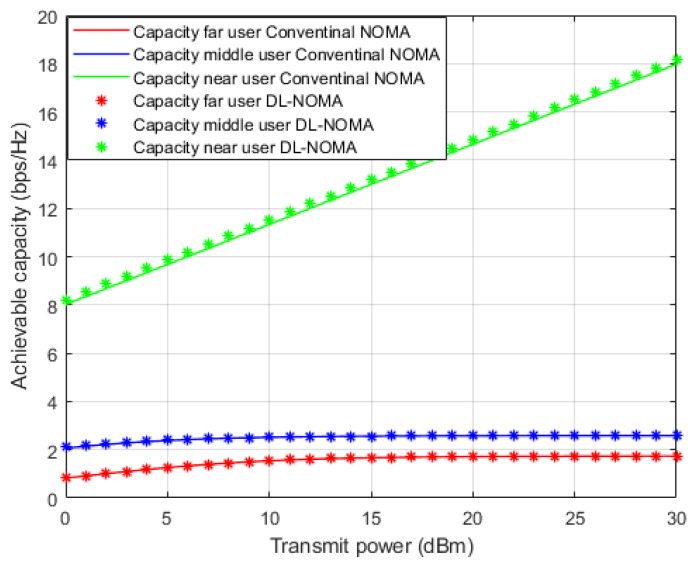
Individual capacity vs. power-based DL and conventional NOMA (Rician Channel).

In Figure 10, the simulation results regarding the individual capacity for each user are illustrated when the Rician channel model is employed, and both the DL-assisted channel estimation and conventional NOMA based on the MMSE channel estimation schemes are applied. It is worth mentioning that for both far and middle users, DL-based channel estimation shows comparable capacity compared to the MMSE scheme, which can be justified, as the DL scheme is not sufficient enough to mitigate the interference and weak channel conditions for far and middle users in the Rician channel. On the other hand, comparable to the Rayleigh fading results, the achieved capacity for the near user shows a significant difference by at least 6 b/s/Hz compared to far and middle users for the same applied power level. This enhancement in capacity can be justified by the line of site component between transmitter and receiver in the Rician channel and the relaxed fading channel between the near user and BS.

**Figure 11:**
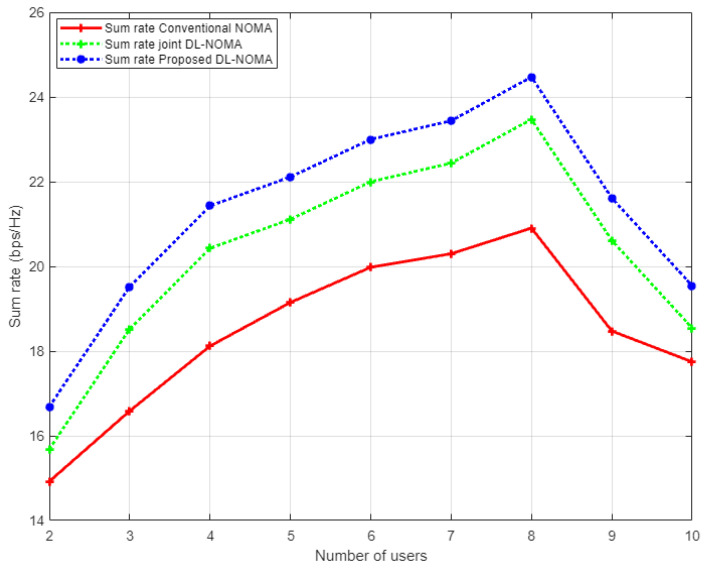
Sum rate vs. number of users for conventional NOMA, joint DL-NOMA, and the proposed DL-NOMA (Rayleigh Channel).

Figure 11 illustrates the simulation results for the sum rate versus the number of users examined in the NOMA cell when the Rayleigh channel model is implemented. In this figure and as a benchmark comparison, we have conducted the simulation environment related to the work in [14], which implements the DL algorithm based on joint channel estimation and signal detection as a one-shot process. As indicated from the figure, our proposed DL-based channel estimation scheme achieves a substantial higher sum rate compared to both the conventional NOMA scheme based on the MMSE procedure, and the DL scheme for joint channel estimation and signal detection discussed in [14]. It can be clearly noticed that as the number of users in the cell increases, our proposed DL channel estimation scheme remains superior in showing higher rates compared to other schemes. These results indicate that reliability can be guaranteed by the proposed scheme even when the cell capacity is increased. On the other hand, it is worth mentioning that as the total number of users keeps increasing in the cell, the interference will also increase, and consequently, the performance will degrade, and the sum rate will start to decrease.

**Figure 12:**
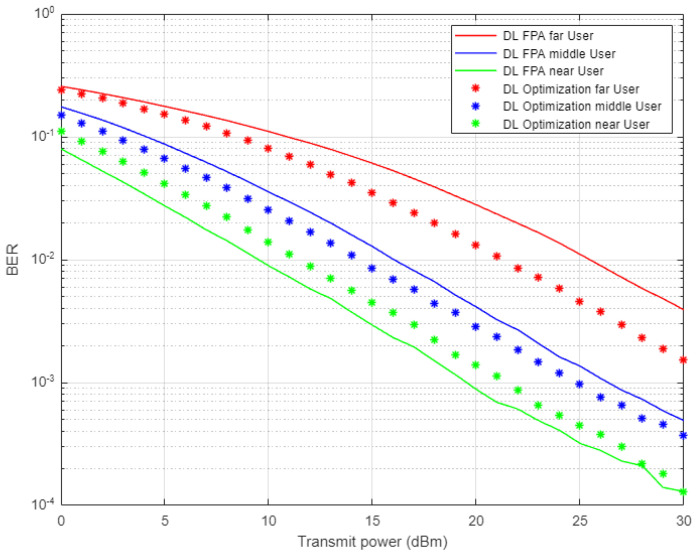
BER vs. power for DL-based optimized and FPA power schemes (Rayleigh Channel).

In Figure 12, two different simulation scenarios are conducted here to generate this figure; the first one when a fixed power allocation (FPA) scheme is applied for each user in the system. The other scenario is the optimized power scheme that is implemented according to the analytical power factors derived earlier. Both scenarios are employed in combination with the proposed DL for the channel estimation scheme. Simulation results for far and middle users prove the superiority of the power-optimized structure over the FPA structure in terms of BER. For the near user results, the proposed DL-based channel estimation jointly with FPA provides little enhancement in terms of the received bits error over the optimized power scheme, which could be justified in that for the near user scenario, a good channel condition is more beneficial than the allocated power.

**Figure 13:**
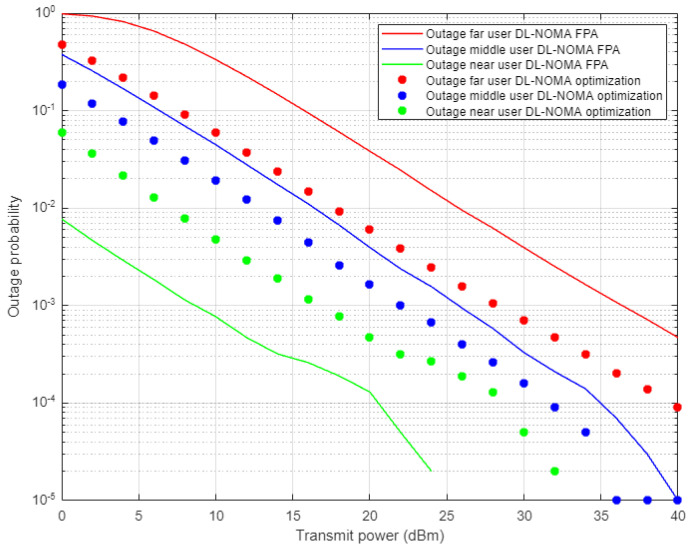
Outage probability vs. power for DL-based optimized and FPA power schemes (Rayleigh Channel).

Figure 13 illustrates the outage probability results against the power transmitted for far, middle, and near users when the optimized power scheme and FPA schemes are applied, and both scenarios are conducted in combination with the proposed DL-based channel estimation in NOMA cell. Far user results show an enhancement in outage probability and the power saving is approximately 5–6 dBm when both the DL and optimized scheme are applied compared to the FPA results. Similarly, for the middle user case, both the DL and optimized scheme provide a noticeable improvement in the outage probability, but with less power saving, i.e., 2–3 dBm approximately. Alternatively, the near user with the joint DL channel estimation scenario and FPA scheme show considerable outage improvement compared to the optimized power case. These results also confirm the results obtained for the outage propagability metric, which indicates that FPA coefficients, jointly with high channel gain, are more sufficient for the near user than the power optimization scheme.

**Figure 14:**
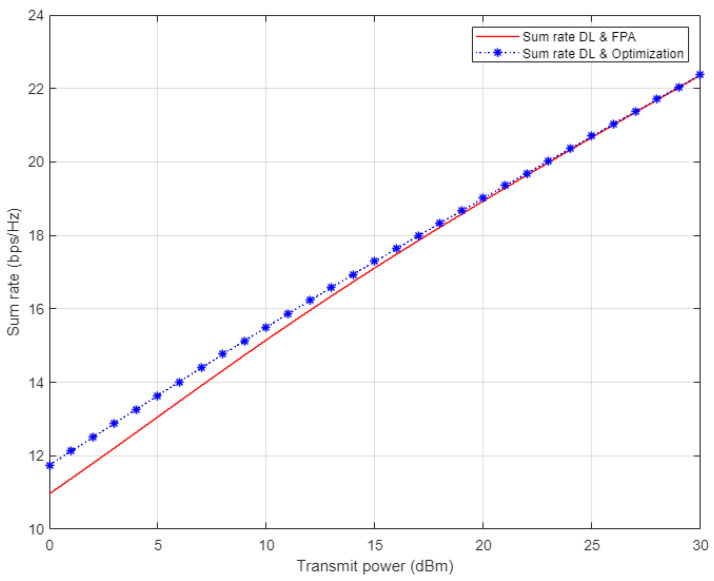
Sum rate vs. power for DL-based optimized and FPA power schemes (Rayleigh Channel).

In Figure 14, the simulation results for the sum rate for the three examined users in NOMA cell are shown. Each of the optimized power scheme and FPA scheme is incorporated with the proposed DL algorithm utilized for estimating the channel coefficients prior to calculating the rate for each user. On the basis of the simulation outcomes, it can be clearly noticed that the channel estimation based on DL combined with the optimized power scheme show little improvement in the sum rate compared to the FPA scenario when the applied power level is low. Starting from 15 dBm, both the optimized power and FPA schemes provide a comparable sum rate when our proposed DL channel estimation scheme is implemented.

**Figure 15:**
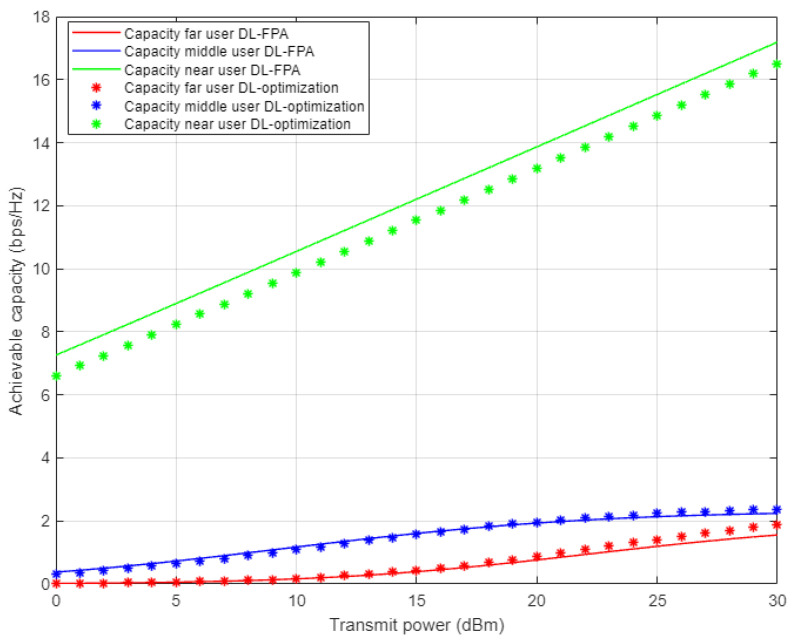
Individual capacity vs. power for DL-based optimized and FPA power schemes (Rayleigh Channel).

In Figure 15, the achievable user’s rates are simulated separately against the transmitted power when the optimized power and fixed power schemes are implemented, where the proposed DL is employed for channel parameter prediction for each user in the cell. The simulation outcomes for far and middle users indicate that both the FPA scheme and the optimized power scheme provide comparable rates, even when the DL algorithm is considered. This might be interpreted as that the control of the power is not always adequate to mitigate the effect of the interference, especially for far and middle users that suffer from fluctuating channel conditions. Unsurprisingly, simulation results for the near user demonstrate dominance in the attainable rate compared to middle and far users by more than 6 b/s/Hz. Additionally, near user results related to fixed power factors show a noticeably better rate compared to the optimized power scheme, which validate the results obtained in Figure 12 and Figure 13, for BER and outage probability metrics, where the FPA scheme revealed visible improvement compared to the optimized power scheme.

## 10. Conclusions and Future Work

In this work, the impact of the Deep Neural Network (DNN) in explicitly estimating the channel coefficients for each user in the NOMA cell is investigated, where the LSTM network is developed for complex data processing. In the proposed DL algorithm, the DNN model is trained online based on both the normalized channel statistics and the relationship between successive training sequences. The validity and efficiency of the proposed DL channel estimation scheme is emphasized by inspecting the proposed DNN model using the Rayleigh fading channel and Rician fading channel. Furthermore, we introduce a framework that investigates how the proposed channel estimation based on the DL and the power optimization scheme are jointly utilized for multiuser detection in the PD-NOMA system. To maximize the sum rate of the system users, we optimize the power coefficients allocated for each user on the basis of the overall power transmitted and the QoS constraints. A systematic mathematical analysis for the optimization problem is introduced and the Lagrange function and KKT conditions are employed to deduce the optimal power factors. The simulation results in terms of the BER, outage probability, sum rate, and individual capacity have verified that the proposed DL model-assisted NOMA can realize reliable performance compared to the conventional NOMA scheme, even when cell capacity is increased.

In future work, the performance of the proposed DNN model can be further explored in terms of single input, multi output (SIMO) or multi input, single output (MISO) for different types of fading channels and modulation schemes.

## Figures and Tables

**Table 1:** Summary of the simulation parameters.

Parameter	Value
Simulation Tool	MATLAB
Modulation type	QPSK
Number of Users	3, [2–10]
System Bandwidth	1 MHz
Fading and channel	(Rayleigh + AWGN), (Rician + AWGN)
No. DNN layers	4
Number of Iterations	10^6^
Optimizer	ADAM
Learning Rate	0.001
Number of neurons/layer (LSTM)	300
Number of pilots	4

## Data Availability

Not applicable.

## Abbreviations

The following abbreviations are used in this manuscript:

AWGN	Additive White Gaussian Noise
BER	bit error rate
BS	Base Station
BTT	Backpropagation through time
CSI	Channel state information
DL	Deep Learning
DNN	Deep Neural Network
FPA	Fixed Power Allocation
GRU	Gated Recurrent Unit
KKT	Karush–Kuhn–Tucker
LSTM	Long Short-Term Memory
LTE	Long Term Evolution
ML	Machine Learning
MSE	Mean Square Error
MMSE	Minimum Mean Square Error
MUD	Multiuser detection
OFDM	Orthogonal Frequency Division Multiplexing
PD-NOMA	Power Domain Non-Orthogonal Multiple Access
QoS	Quality of Service
RBM	Restricted Boltzmann Machines
RNN	Recurrent Neural Networks
SIC	Successive interference cancellation
SIMO	Single-input, multi-output
